# Dual-energy CT-based nomogram for predicting progression-free survival in locally advanced nasopharyngeal carcinoma

**DOI:** 10.3389/fonc.2025.1698927

**Published:** 2025-11-20

**Authors:** Wei Pei, Chen Wang, Yunyun Wei, Jisheng Xie, Danke Su, Hai Liao

**Affiliations:** 1 Department of Radiology, Guangxi Medical University Cancer Hospital, Nanning, China; 2 Department of Nuclear Medicine, Guangxi Medical University Cancer Hospital, Nanning, China

**Keywords:** dual-energy CT, nasopharyngeal carcinoma, nomogram, progression-free survival, prognosis

## Abstract

**Purpose:**

To establish a dual-energy CT (DECT) based nomogram for predicting progression-free survival (PFS) in locally advanced nasopharyngeal carcinoma (LANPC).

**Methods:**

In this retrospective study, 52 LANPC patients who underwent DECT scans and post-treatment follow-up (median follow-up = 42.2 months) were enrolled. DECT parameters of tumor lesions including iodine concentration (IC), normalized iodine concentration (NIC), the slope of the spectral Hounsfield unit (HU) curve (λ_HU_), and effective atomic number (Zeff) were analyzed to predict PFS. A nomogram integrating clinical data and DECT-derived parameters was constructed. The model’s performance was evaluated using calibration curves, Harrell’s concordance index (C-index), and receiver operating characteristic (ROC) curve.

**Results:**

NIC, neutrophil-to-lymphocyte ratio (NLR), and lactate dehydrogenase (LDH) were the independent prognostic factors for PFS, and were incorporated into constructing the nomogram. Calibration plots demonstrated strong agreement between predicted and observed PFS rates. The C-index for the nomogram was 0.88 (95% confidence interval [CI]: 0.80–0.90). The nomogram model demonstrated predictive accuracy for PFS, with the area under the ROC curves (AUCs) of 0.939, 0.880, and 0.879 at 1-, 2-, and 3-year, respectively.

**Conclusion:**

The DECT-based nomogram exhibited excellent predictive accuracy for PFS in LANPC patients, highlighting its potential as a valuable clinical tool.

## 1 Introduction

Nasopharyngeal carcinoma (NPC) is an endemic malignancy predominantly found in Southeast Asia and southern China (1). In 2020, among the 133,354 newly diagnosed NPC cases globally, over 70% were classified as locoregionally advanced NPC (LANPC) (2). The National Comprehensive Cancer Network (NCCN) guidelines recommend induction chemotherapy (ICT) combined with concurrent chemoradiotherapy (CCRT) as the first-line treatment for LANPC (3). With the implementation of successful radiotherapy and chemotherapy strategies, the 5-year survival rate of LANPC patients has significantly improved (4). Despite these advancements, approximately 30% of patients still develop recurrent or metastatic disease, which remains the leading cause of mortality in NPC (5). Therefore, accurate pretreatment prediction of prognosis in LANPC patients is critical to guide clinicians in formulating personalized treatment plans.

Extensive research has been conducted to identify effective biomarkers for predicting prognosis in patients with locally advanced nasopharyngeal carcinoma. These biomarkers include plasma Epstein-Barr virus (EBV) DNA (6) and various imaging modalities, such as positron emission tomography/computed tomography (PET/CT) (7), PET/MRI (8), dynamic contrast-enhanced (DCE)-MRI (9), and intravoxel incoherent motion (IVIM) imaging (10).The clinical applicability of EBV-DNA has been constrained by variations in institutional reference standards and testing protocols. Nevertheless, PET/CT is limited by factors including restricted availability and high cost. Both DCE-MRI and IVIM imaging necessitate advanced equipment and time-intensive post-processing, which has limited their widespread adoption. Recent studies have investigated machine learning and deep learning-based radiomics models for prognostic prediction in nasopharyngeal carcinoma; however, these approaches frequently involve complex models and cumbersome data analysis (11, 12). Consequently, an unmet need persists for a direct and accurate method of prognostic stratification. CT continues to be essential for the assessment of bone invasion in nasopharyngeal carcinoma (13). Dual-energy CT (DECT) has emerged as a valuable tool for evaluating tumor angiogenesis, cellular density, and treatment response by enabling quantitative analysis of functional parameters such as iodine concentration (IC), normalized iodine concentration (NIC), and effective atomic number (Zeff) (14–16). Studies have demonstrated that DECT can accurately quantify iodine content, serving as a quantitative biomarker for tissue angiogenesis and cellular density, both of which are critical prognostic factors in gastric and breast cancers (17, 18). However, an integrated predictive model combining DECT parameters with clinical indicators has yet to be developed.

It is hypothesized that integrating the DECT parameter (NIC) with clinical biomarkers will enhance the prediction of progression-free survival (PFS) beyond that achievable by clinical factors alone. To test this hypothesis, a novel nomogram combining DECT functional parameters with clinical indicators will be developed to predict PFS in patients with LANPC. The multiparameter model is expected to provide a more precise and comprehensive prognostic assessment tool for clinical application.

## 2 Materials and methods

### 2.1 Patient data

**Figure 1:**
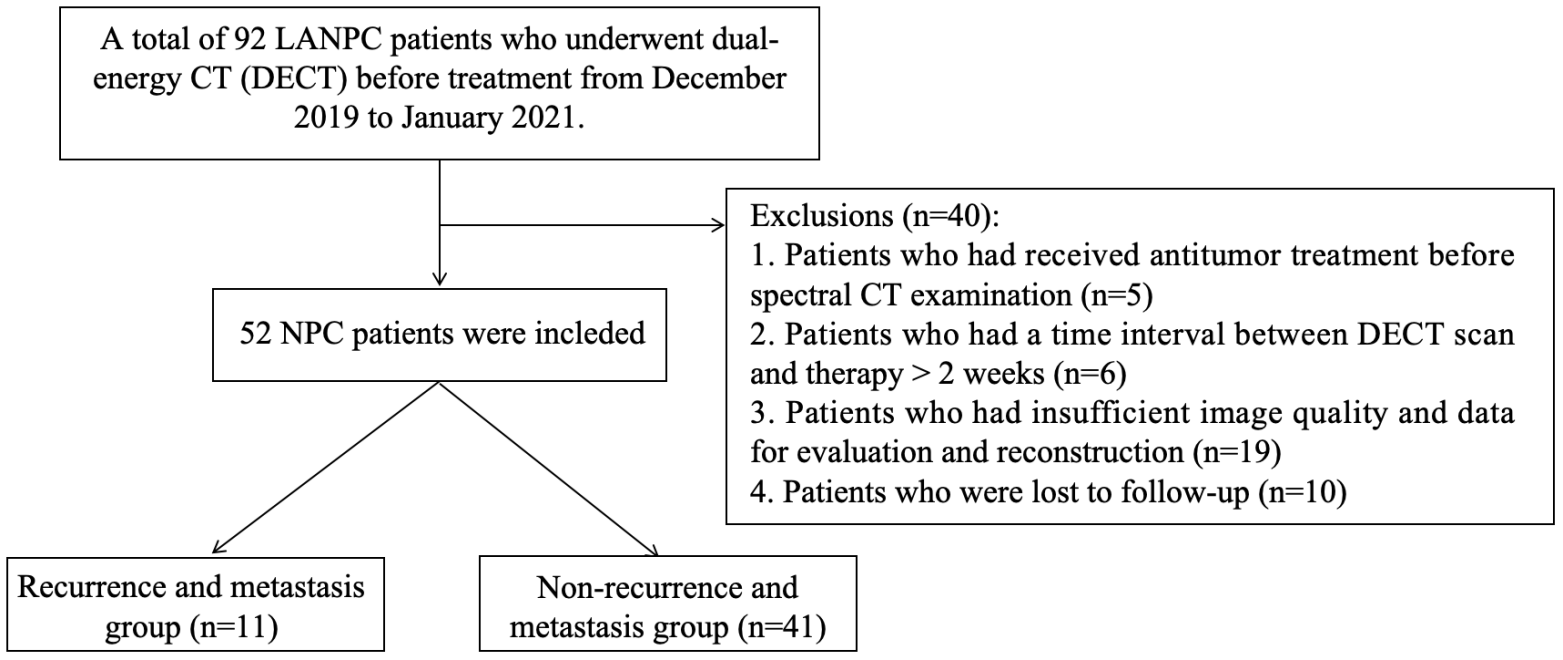
Patient selection flowchart.

This study was approved by our institutional review board, and the need for patients’ informed consent was waived due to the retrospective nature of this study. A total of 52 LANPC patients from December 2019 to January 2021 were consecutively enrolled in our study. As shown in **Figure 1**, recruitment was based on the following inclusion criteria (1): nonkeratinizing squamous cell carcinoma confirmed by histopathology and (2) pretreatment DECT was performed; and (3) stage III to IVa disease (according to American Joint Committee on Cancer [AJCC] 8th head and neck tumor staging criteria) (19) were scheduled for ICT+CCRT. LANPC patients were excluded if they (1) had received antitumor treatment before DECT examination (2); had a time interval between DECT scan and therapy > 2 weeks (3); had insufficient image quality and data for evaluation and reconstruction; or (4) were lost to follow-up.

Demographic and clinical data (including age, gender, smoking status, family history, T stage, N stage, clinical stage, body mass index [BMI], ICT response, pathological type, white blood cell [WBC], red blood cells [RBC], platelet [PLT], neutrophil [NEUT], lymphocyte [LYM], albumin [ALB], lactate dehydrogenase [LDH], and neutrophil-to-lymphocyte ratio [NLR])were collected.

### 2.2 Treatment and data collection

Induction chemotherapy combined with concurrent chemoradiotherapy was administered to all enrolled patients. The specific treatment regimen is described as follows: For ICT, the TPF regimen was administered as docetaxel (60 mg/m² intravenously on days 1, 22, and 43), cisplatin (60 mg/m² intravenously on days 1, 22, and 43), and fluorouracil (600 mg/m²/day via continuous 120-hour infusion on days 1–5, 22–26, and 43–47), with cycles repeated every 3 weeks. The GP regimen consisted of gemcitabine (1 g/m² intravenously on days 1, 8, 22, 29, 43, and 64) and cisplatin (80 mg/m² intravenously on days 1, 2, 22, 23, 43, and 44), also delivered in 3-week cycles. Both regimens were administered for three cycles with 21-day intervals between each cycle. CCRT included cisplatin (40 mg/m² daily for 5 consecutive days per week, repeated every 3 weeks for a total of three cycles), initiated on the first day of radiotherapy. Intensity-modulated radiotherapy (IMRT) was delivered with a fractionated schedule: the primary tumor received 66–72 Gy in 30–33 fractions (2.0–2.4 Gy per fraction), while the prophylactic irradiation area received 54–56 Gy in 30 fractions (1.8-1.9 Gy per fraction).

Patients were followed up every 1-3months in the first 2 years, once every 6 months in the 3-5years, and once a year thereafter. All participants were followed up for at least 2 years. The study endpoint was the PFS, which was calculated from the starting of treatment to the disease progression (or censored at the last follow-up).

### 2.3 Recurrence and metastasis group and follow-up

Based on the follow-up results, patients were categorized into two groups: the recurrence and metastasis group and the non-recurrence and metastasis group. The definitions of NPC recurrence and distant metastasis were as follows (1) Recurrence: In patients with a pathological diagnosis of NPC, the clinical tumor was completely eradicated following radical treatment. However, a tumor of the same pathological type as the primary tumor reappeared in the local or cervical lymphatic drainage area six months or more after the completion of treatment, which was diagnosed as recurrence (2) Distant Metastasis: No distant metastasis was detected at the initial diagnosis. However, post-treatment imaging revealed single or multiple metastases in the lungs, liver, brain, bones, axillary lymph nodes, chest wall, or multiple organs, confirmed by pathological biopsy. In cases where pathological biopsy was unavailable, distant metastasis was diagnosed based on clinical history, tumor markers, and the exclusion of primary tumors in other organs.

### 2.4 DECT protocol

All examinations were conducted using the Discovery CT750 CT scanner (GE Healthcare). The scans covered the area from the base of the skull to the thoracic inlet. The scanning parameters were set as follows: dual-energy acquisition at 80/140 kVp, tube current of 360/375 mA, rotation time of 0.7 seconds, detector collimation of 4 cm, and Adaptive Statistical Iterative Reconstruction (ASIR-V) technology with a 40% weighting factor. Additional parameters included a pitch of 0.984:1, slice thickness of 5 mm, and reconstruction interval of 5 mm.

The imaging protocol began with a localization scan, followed by a conventional spiral scan. After this, 70 mL of iodinated contrast agent (300 mgI/mL) and 20 mL of normal saline were administered through the antecubital vein at a rate of 3.0 mL/s. Enhanced scans were then performed using energy spectrum CT, with the arterial phase acquired at a 25-second delay and the venous phase at a 50-second delay post-injection.

### 2.5 DECT image analysis

All images were transferred to the Gemstone Spectral Imaging (GSI) Viewer 4.6 workstation for image analysis and post-processing. Two head and neck radiologists (with 5 and 18 years of experience, respectively) independently analyzed the images while blinded to clinical information, delineating regions of interest (ROIs) based on lesion boundaries.

To ensure consistency and reproducibility, ROIs segmentation were conducted following a standardized operating procedure (SOP). For each patient, the two-dimensional (2D) ROIs were manually delineated on the axial venous phase images. For each parameter, three independent ROIs were selected for triplicate measurements, and the average value was calculated. Subsequently, the mean value from both observers was obtained. ROIs were carefully positioned to avoid areas of cystic necrosis, calcification, prominent vasculature, and artifacts, while ensuring maximal lesion coverage and consistent area across measurements.

All DECT parameters for the final model were derived from the venous phase scans, as this phase provides a more stable assessment of tumor iodine uptake and has been widely used in previous oncology studies. For the same axial slice, the IC of the nasopharyngeal lesion and the carotid artery iodine concentration (IC-C) were measured, along with the Zeff. The NIC and the slope of the spectral Hounsfield unit (HU) curve (λ_HU_) were calculated using the following formulas:


$$\text{NIC }=\frac{\text{IC}}{\text{ IC}-\text{C}}$$

$${\text{λ}}_{\text{HU}}\;=\frac{\text{CT}40\text{keV }–\text{ CT}100\text{keV}}{\;60}$$

where CT40keV and CT100keV represent the CT values of the ROI under 40 keV and 100 keV monochromatic energy levels, respectively.

### 2.6 Statistical analysis

Categorical variables were compared using the Chi-square test or Fisher’s exact test, while continuous variables were analyzed with the Mann-Whitney U test. The optimal cut-off value for the continuous variable NLR was determined using maximally selected log-rank statistics (via the ‘maxstat’ package in R) to identify the threshold that best discriminated between PFS outcomes. This data-driven approach yielded a cut-off value of NLR≥3, which was subsequently dichotomized for inclusion in the regression models.

The variable selection process for the Cox proportional hazards model was conducted as follows: Initially, univariate Cox regression analyses were performed on all potential predictors, including clinical, laboratory, and DECT parameters. Variables demonstrating a *P*-value less than 0.1 in the univariate analysis were subsequently included in the multivariate Cox regression analysis. Prior to the multivariate analysis, variance inflation factors (VIFs) were calculated to evaluate multicollinearity; a VIF below 5 was deemed acceptable, indicating the absence of significant collinearity among predictors. The final multivariate model was developed using a backward stepwise selection procedure guided by the Akaike Information Criterion (AIC), thereby identifying the most parsimonious model with optimal fit.

Given the limited number of events, internal validation of the final nomogram was performed using bootstrap resampling with 1000 iterations. The optimism-corrected C-index was calculated to provide a more robust estimate of the model’s discriminative performance and to correct for any overfitting.

Subsequently, a nomogram was constructed based on the results of the multivariate Cox regression analysis. The predictive accuracy and discriminative ability of the nomogram were evaluated using calibration curves, Harrell’s concordance index (C-index), and area under the curve (AUC).

All statistical analyses were conducted using SPSS version 26.0 (SPSS Inc., Chicago, IL, USA) and R version 3.6.3 (The R Foundation for Statistical Computing, Vienna, Austria; https://www.r-project.org/). A two-sided p-value < 0.05 was considered statistically significant.

## 3 Results

### 3.1 Patient clinical factors and DECT parameters

**Figure 2:**
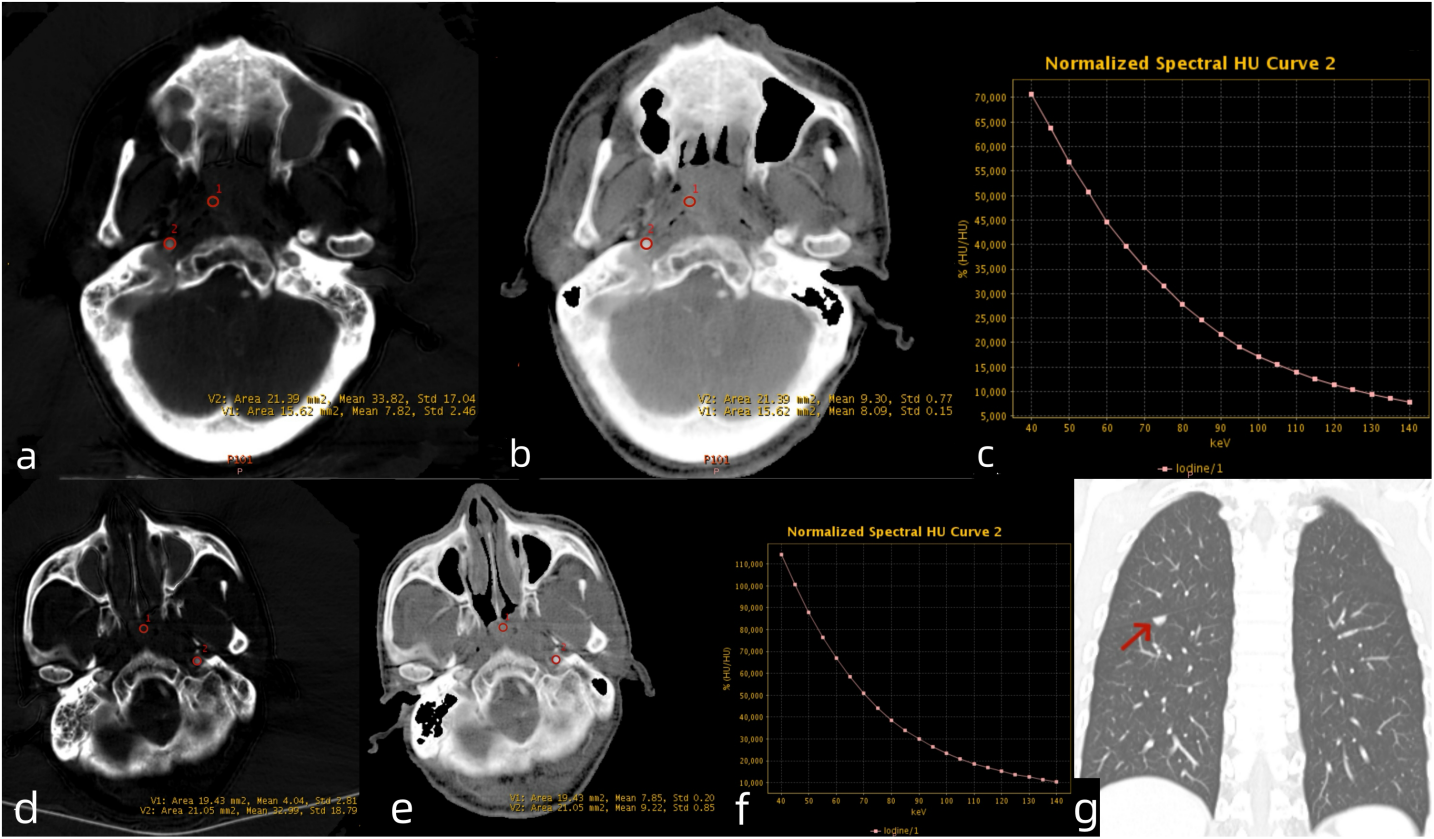
Venous phase DECT images of LANPC patients. A 61-year-old male patient from the non-recurrence and metastasis group. **(a)** Iodine-based material decomposition image, **(b)** Zeff map, **(c)**DECT λ_HU_ map. The nasopharyngeal lesion demonstrated IC = 7.82 mg/mL, IC-C = 33.82 mg/mL, NIC = 0.23, Zeff = 8.09, and λ_HU_ = 0.88. A 54-year-old male patient from the recurrence and metastasis group. **(d)** Iodine-based material decomposition image, **(e)** Zeff map, **(f)** DECT λ_HU_ map and **(g)** coronal chest CT image. The nasopharyngeal lesion demonstrated IC = 4.04 mg/mL, IC-C = 32.99 mg/mL, NIC = 0.13, Zeff = 7.85, and λ_HU_ = 0.91. The patient developed lung metastases 6 months after treatment completion. LANPC = locally advanced nasopharyngeal carcinoma; IC = iodine concentration; IC-C = carotid artery iodine concentration; NIC = normalized iodine concentration.

The clinical factors and DECT parameters of patients are detailed in **Table 1**. A total of 52 patients were included in this study, comprising 43 male and 9 female patients, with an age range of 22 to 68 years. **Table 1** shows the detailed clinical characteristics of the patients. The median follow-up time was 42.2 months. Among the 43 male patients, 8 (18.6%) experienced recurrence, while 3 (33.3%) of the 9 female patients had a recurrence. The 1-year, 2-year, and 3-year PFS rates were 94.4%, 88.9%, and 66.7%, respectively. Univariate analysis identified ICT response, LDH, NLR, IC, NIC and Zeff as significant predictors of PFS (all *P* < 0.05). The DECT parameters for the non-recurrent metastasis group and the recurrent metastasis group are presented in **Figure 2**. No significant statistical differences were observed between recurrence and metastasis group and non-recurrence and metastasis group in age, gender, smoking status, family history, T/N stage, clinical stage, pathological classification, WBC, RBC, PLT, NEUT, LYM, ALB, and λ_HU_.

**Table 1:** Clinical characteristics and DECT parameters of the patients.

Variables	Recurrence and metastasis group (n=11)	Non-recurrence and metastasis group (n=41)	*P*-value
Age(years)	52.82 ± 11.75	48.93± 10.34	0.286
Gender			0.554
Male	8 (72.7%)	35 (85.4%)	
Female	3 (27.3%)	6 (14.6%)	
Smoking status			0.561
Yes	6 (54.5%)	25 (60.9%)	
No	5 (45.4%)	16 (39.1%)	
BMI	21.96 ± 1.84	22.71 ± 3.18	0.670
Family history			0.191
Yes	3 (27.3%)	3 (7.3%)	
No	8 (72.7%)	38(92.7%)	
T stage			0.345
T1	0 (0)	2 (4.9%)	
T2	0 (0)	8(19.5%)	
T3	6 (54.5%)	17(41.5%)	
T4	5 (45.4%)	14 (34.1%)	
N stage			0.708
N0	0 (0)	2(4.9%)	
N1	6 (54.5%)	14 (34.1%)	
N2	2 (18.2%)	12(29.3%)	
N3	3 (27.3%)	12 (29.3%)	
N4	0(0)	1(2.4%)	
Clinical stage			0.714
III	3 (27.3%)	16(39.1%)	
IVa	8 (72.7%)	25 (60.9%)	
ICT response			0.001*
Response	5(45.4%)	38 (92.7%)	
Non-response	6 (54.5%)	3(7.3%)	
Pathological type			0.669
Keratinized type	0 (0)	0 (0)	
Non-keratinized differentiation type	1(9.1%)	2 (4.9%)	
Non-keratinized and undifferentiated	10 (90.9%)	37(90.2%)	
Undifferentiated + differentiated mixed type	0 (0)	2 (4.9%)	
WBC (×10^9^/L)	7.52 ± 1.66	6.96 ± 1.78	0.359
RBC(×10^9^/L)	134.73 ± 9.42	138.15 ± 15.44	0.230
PLT(×10^9^/L)	265.36 ± 74.59	249.78 ± 64.26	0.493
NEUT(×10^9^/L)	4.79 ± 1.35	4.21 ± 1.30	0.198
LYM(×10^9^/L)	3.33 ± 5.34	1.85 ± 0.71	0.084
ALB(×10^9^/L)			0.604
LDH(U/L)	208.18 ± 49.93	171.89 ± 36.37	0.009*
NLR			0.001*
<3	3 (27.3%)	35 (85.4%)	
≥3	8 (72.7%)	6 (14.6%)	
DECT parameters			
IC (100 mg/mL)	5.62 ± 2.69	7.52 ± 6.13	0.037*
NIC	0.21 ± 0.19	0.31 ± 0.10	0.004*
Zeff	7.92 ± 0.19	8.05 ± 0.16	0.029*
λ_HU_	0.64 ± 0.19	0.54 ± 0.16	0.078

*Statistically significant variables with a *P*-value < 0.05.

DECT, dual-energy CT; BMI, body mass index; ICT, induction chemotherapy; WBC, white blood cell; RBC, red blood cells, PLT, platelet; NEUT, neutrophil; LYM, lymphocyte; ALB, albumin; LDH, lactate dehydrogenase; NLR, neutrophil-to-lymphocyte ratio; IC, iodine concentration; NIC, normalized iodine concentration; Zeff, effective atomic number; λ_HU_, slope of spectral HU curve.

### 3.2 Multivariate Cox regression analysis

Multivariate Cox regression analysis identified three independent prognostic factors, as summarized in **Table 2**:

**Table 2:** Univariate and multivariate Cox analyses for PFS of LANPC patients.

Variables	Univariate analysis		Multivariate analysis	
	HR (95%CI)	*P*-value	HR (95%CI)	*P*-value
ICT response	7.123(2.145-23.658)	0.001^*^	1.177(0.830-52.928)	0.074
LDH	1.016 (1.004-1.028)	0.008^*^	1.020(1.010-1.030)	0.030^*^
NLR	7.60 (1.640-35.320)	0.010^*^	5.510(1.140-26.630)	0.034^*^
IC	0.809(0.642-1.018)	0.071	1.177(0.493-2.815)	0.715
NIC	0.000(0.000-0.212)	0.014^*^	0.000(0.000-0.500)	0.032^*^
Zeff	0.038(0.001-1.159)	0.061	0.764(0.000- 24121.4)	0.959

*Statistically significant variables with a *P*-value < 0.05.

ICT, induction chemotherapy; LDH, lactate dehydrogenase; NLR, neutrophil-to-lymphocyte ratio; IC, iodine concentration; NIC, normalized iodine concentration; Zeff, effective atomic number.


LDH: Hazard ratio (HR) = 1.02 (95% confidence interval [CI]: 1.010–1.030, P = 0.030)

NLR: HR = 5.51 (95% CI: 1.14–26.63, P = 0.034)

NIC: HR = 0.22(95% CI: 0.06–0.79, P = 0.032)

### 3.3 Construction and validation of the nomogram

**Figure 3:**
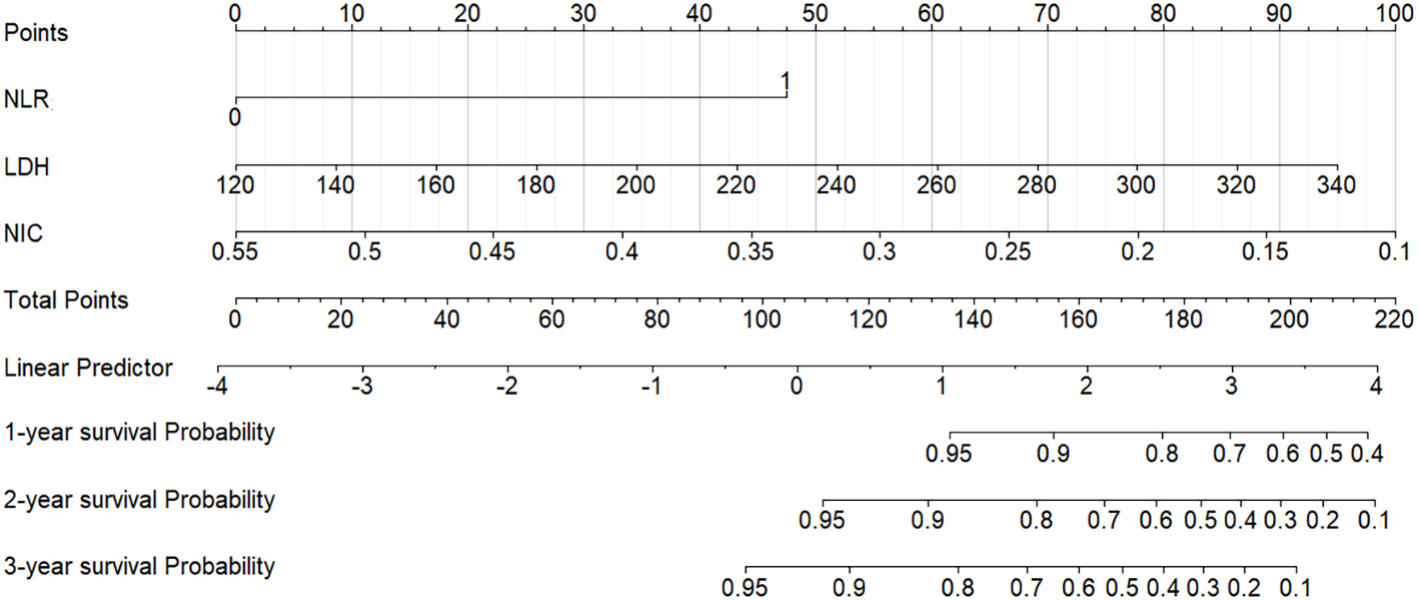
Nomogram for predicting 1-year, 2-year, and 3-year PFS in LANPC patients. Points are assigned for each prognostic factor (NIC, NLR, LDH), and total points correspond to predicted survival probabilities. PFS = progression-free survival; LANPC = locally advanced nasopharyngeal carcinoma; NIC = normalized iodine concentration; NLR = neutrophil-to-lymphocyte ratio; LDH = lactate dehydrogenase.

**Figure 4:**
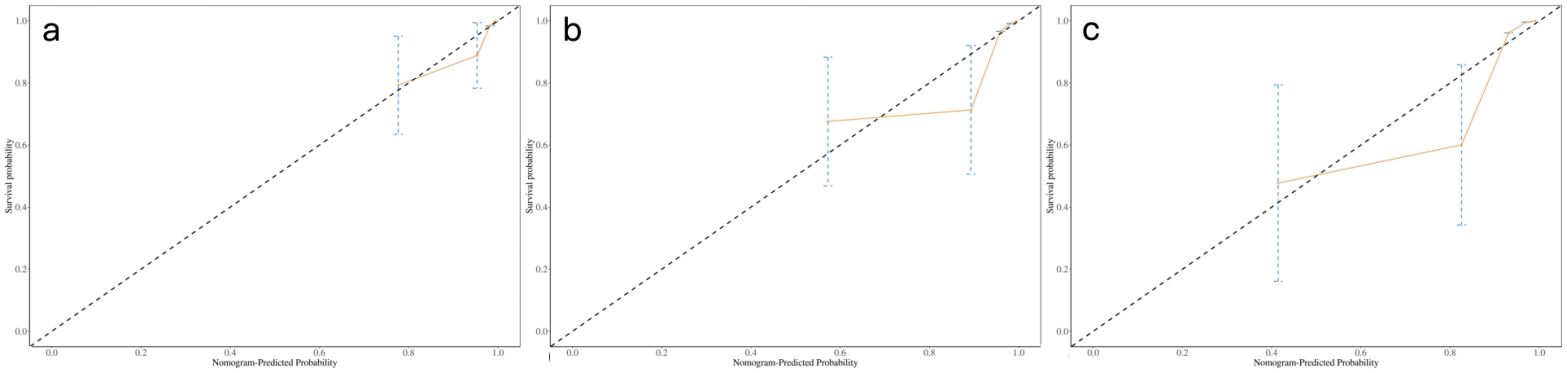
Calibration curves of nomogram for predicting 1-, 2-, and 3-year probability of PFS **(a-c)**. Actual PFS is plotted on the y-axis; nomogram- predicted probability of PFS is plotted on the x-axis. PFS = progression-free survival.

**Figure 5:**
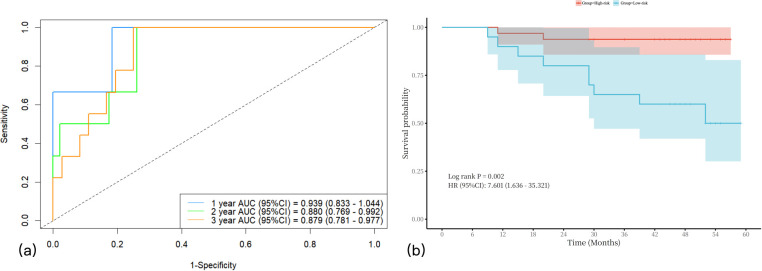
The AUCs of nomogram in predicting 1-year, 2-year, and 3-year PFS in LANPC patients **(a)**. Survival analysis stratified by the nomogram model **(b)**. AUCs = area under the curves; LANPC = locally advanced nasopharyngeal carcinoma.

A nomogram integrating NIC, NLR, and LDH was developed to predict 1-year, 2-year, and 3-year PFS probabilities (**Figure 3**). The model demonstrated excellent discriminative ability, with a Harrell’s C-index of 0.88 (95% CI: 0.79–0.97). The calibration curves for 1-, 2- and 3-year PFS rates were largely overlapped with the standard lines (**Figure 4**). To evaluate the performance of the nomogram, the ROC curves were generated, and the AUC were calculated. In ROC analysis, the AUCs of the nomogram for 1-, 2- and 3-year PFS prediction were 0.939, 0.880 and 0.879, respectively (**Figure 5a**).

### 3.4 Kaplan-Meier curves

To further visualize the discriminatory power of this predictive model, the total risk score for each patient was calculated based on the final Cox model. Subsequently, patients were stratified into low-risk and high-risk groups according to these risk scores. Kaplan-Meier survival curves with a risk table were generated (**Figure 5b**), and the log-rank test confirmed significant differences in PFS among the two risk groups (*P* < 0.001).

## 4 Discussion

This study innovatively integrates DECT function parameters with clinical factors, including NLR and LDH, to develop a nomogram for predicting PFS in patients with LANPC. Multivariate Cox regression analysis revealed that NIC, NLR, and LDH were independent prognostic factors for PFS. The constructed nomogram demonstrated excellent predictive performance, with a C-index of 0.88 and well-fitted calibration curves. Significant differences in PFS between low-risk and high-risk groups, as defined by the nomogram, were demonstrated by Kaplan–Meier survival analysis.

The novelty of this method resides in the application and evaluation of DECT parameters, namely IC, NIC, λ_HU_, and Zeff, for prognostic prediction in LANPC. Although conventional CT remains the standard for anatomical staging, the utility of DECT is extended by its ability to provide functional and compositional information. IC and its normalized counterpart, NIC, quantify tumor blood volume and perfusion status, thereby reflecting angiogenesis. Additionally, λ_HU_ contributes to tissue characterization, while Zeff provides an estimate of the tumor’s elemental composition. While these parameters have been previously utilized in other malignancies for lesion characterization and assessment of treatment response (20, 21), their comprehensive evaluation and incorporation into prognostic models for NPC, especially within the context of chemoradiotherapy following induction chemotherapy, has been limited. The present preliminary analysis of all four parameters constitutes a thorough investigation. Notably, multivariate modeling revealed NIC to be the most robust imaging predictor, surpassing IC, λ_HU_, and Zeff. This finding suggests that NIC may exhibit greater biological significance and statistical robustness for prognostic modeling in this clinical scenario. This is consistent with previous findings in solid tumors (22). As a standardized ratio parameter reflecting tumor vascular heterogeneity, may derive its predictive advantage from correcting for individual circulatory variations (23). A low NIC value indicates that the IC of tumor neovascular is lower than that of mature carotid artery vasculature. This imbalance may promote recurrence and metastasis through the following mechanisms (1): Abnormal vascular permeability leading to interstitial hypertension, impeding chemotherapeutic drug penetration (24) (2): Inefficient perfusion exacerbating tumor hypoxia, stimulating cancer cell proliferation and invasiveness, while reducing sensitivity to radiotherapy and chemotherapy (25). However, these findings appear to contradict the study by Zhan et al. (26), which proposed that elevated NIC values serve as an independent predictor of poor survival in NPC. Their survival analyses indicated that NPC patients with higher NIC values in primary tumors often exhibited worse prognosis compared to those with lower values. The discrepancies between these studies may arise from several technical and biological factors. Technically, variations in the timing of contrast phases (arterial versus venous) used for quantification significantly influence absolute iodine values. Biologically, the relationships among perfusion, hypoxia, and prognosis are complex: while moderate vascularization facilitates drug delivery, excessive and disorganized angiogenesis may cause uneven perfusion and hypoxia. Additionally, patient heterogeneity, including regional variations in NPC incidence, individual characteristics, and treatment diversity, may further explain the observed discrepancies.

This study corroborates prior observations that NLR(≥3) and LDH levels are associated with disease recurrence in patients with LANPC. An elevated NLR serves as a marker of systemic inflammatory response. Previous studies have linked elevated NLR with an immunosuppressive tumor microenvironment and an increased risk of distant metastasis (27, 28). Elevated pretreatment LDH levels have also been confirmed as prognostic indicators of poorer outcomes in NPC patients (29). LDH, a key enzyme in glycolysis, facilitates tumor cell adaptation to hypoxic microenvironments. Its elevation correlates closely with tumor burden, invasiveness, and resistance to treatment (30).

Compared to models relying solely on imaging or clinical parameters, the integrated multiparametric model significantly enhances predictive accuracy. DECT-derived NIC provides direct insights into local tumor biology, while NLR) and LDH offer complementary information regarding systemic inflammation and metabolism. It is imperative that this model be evaluated within the context of the existing prognostic framework for NPC, a domain historically dominated by established plasma Epstein-Barr virus DNA (EBV-DNA) biomarkers (31). Given that EBV-DNA remains a robust prognostic indicator, comparative assessment with any novel prognostic model is warranted. The present study concentrated on developing models based on readily accessible clinical data and DECT parameters. While the exclusion of EBV-DNA data represents a limitation, it delineates the study’s scope by focusing on the prognostic value of imaging-centric models with high accessibility. Future investigations directly comparing the incremental value of DECT parameters relative to EBV-DNA are advised and represent a critical direction for subsequent research. Moreover, in contrast to advanced MRI techniques, such as dynamic contrast-enhanced MRI or hemodynamic imaging, or PET/CT, which require additional, time-intensive sequences, specialized protocols, or separate appointments, DECT parameters can be derived directly from contrast-enhanced CT scans without prolonging examination time or substantially increasing radiation exposure. Although the initial capital expenditure for DECT scanners is considerable, the long-term operational costs approximate those of conventional CT systems. The potential cost-effectiveness of this predictive model lies in its capacity to improve risk stratification accuracy during initial staging. By identifying patients at high risk who may benefit from intensified treatment or vigilant surveillance, and those at low risk who might avoid unnecessary overtreatment, the model facilitates optimized allocation of healthcare resources. This strategy holds promise for enhancing overall oncological outcomes, thereby compensating for the initial technological investment.

However, this study has several limitations. First, the statistical power was limited by the relatively small sample size (n=52) derived from a single institution, which also increased the risk of model overfitting. Second, all imaging was conducted using a single type of CT scanner from one vendor, and the manual, although meticulous, ROI delineation method, without formal reporting of the inter-observer intraclass correlation coefficient (ICC), may limit the generalizability and reproducibility of the results. To enhance robustness, future studies are encouraged to employ semi-automated or volumetric segmentation techniques. Third, despite all patients receiving ICT followed by CCRT, heterogeneity existed in the chemotherapy regimens administered, which may have influenced clinical outcomes. Most importantly, the predictive model was developed and internally validated solely via bootstrap resampling. The absence of an external validation cohort, either temporally or geographically distinct, precludes confirmation of the nomogram’s true performance and generalizability. Collectively, these limitations underscore the preliminary nature of the findings and emphasize the need for future multi-center, large-scale prospective studies incorporating external validation.

## 5 Conclusions

This preliminary study demonstrates that a nomogram integrating NIC, NLR, and LDH shows potential for predicting PFS in patients with LANPC. By combining quantitative imaging with conventional clinical biomarkers, the model constitutes a significant advancement toward personalized prognostic prediction. Nevertheless, due to the aforementioned limitations, specifically, the single-center retrospective design and limited sample size, these findings should be regarded as exploratory. Future research is warranted to incorporate longitudinal data, external validation cohorts, and additional radiological features in order to develop robust, dynamic prognostic models and to further assess their clinical utility for optimizing personalized treatment strategies.

## Data Availability

The raw data supporting the conclusions of this article will be made available by the authors, without undue reservation.
